# Efficient Removal of Tetracycline and Bisphenol A from Water with a New Hybrid Clay/TiO_2_ Composite

**DOI:** 10.1021/acsomega.3c00184

**Published:** 2023-06-05

**Authors:** Morenike O. Adesina, Inga Block, Christina Günter, Emmanuel I. Unuabonah, Andreas Taubert

**Affiliations:** † Institute of Chemistry, University of Potsdam, D-14476 Potsdam, Germany; ‡ African Centre of Excellence for Water and Environment Research (ACEWATER), Redeemer’s University, PMB 230 Ede, Osun State 232101, Nigeria; § Department of Chemical Sciences, Redeemer’s University, PMB 230 Ede, Osun State 232101, Nigeria; ∥ Lead City University, Ibadan 200255, Oyo State, Nigeria; ⊥ Institute of Geosciences, University of Potsdam, D-14476 Potsdam, Germany

## Abstract

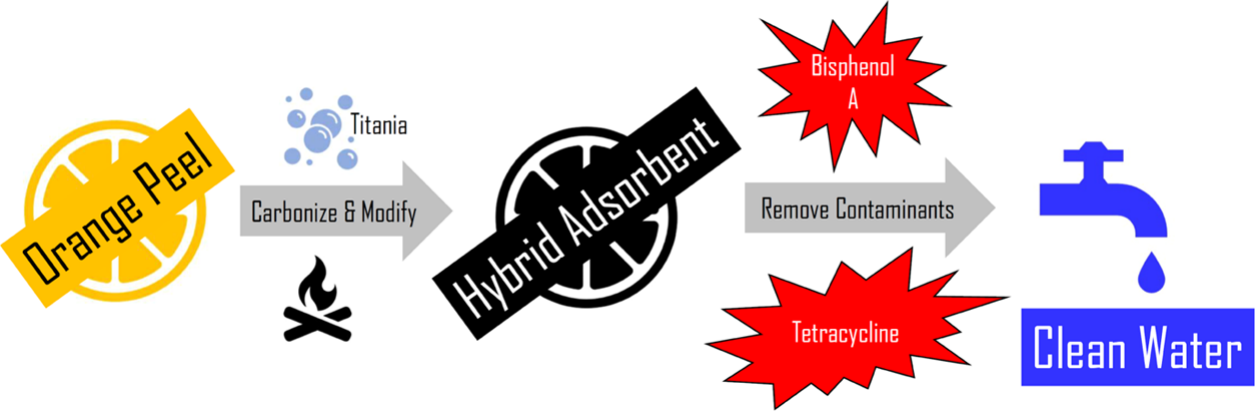

New TiO_2_ hybrid composites were prepared from kaolin clay, predried and carbonized biomass, and titanium tetraisopropoxide and explored for tetracycline (TET) and bisphenol A (BPA) removal from water. Overall, the removal rate is 84% for TET and 51% for BPA. The maximum adsorption capacities (*q*_m_) are 30 and 23 mg/g for TET and BPA, respectively. These capacities are far greater than those obtained for unmodified TiO_2_. Increasing the ionic strength of the solution does not change the adsorption capacity of the adsorbent. pH changes only slightly change BPA adsorption, while a pH > 7 significantly reduces the adsorption of TET on the material. The Brouers–Sotolongo fractal model best describes the kinetic data for both TET and BPA adsorption, predicting that the adsorption process occurs via a complex mechanism involving various forces of attraction. Temkin and Freundlich isotherms, which best fit the equilibrium adsorption data for TET and BPA, respectively, suggest that adsorption sites are heterogeneous in nature. Overall, the composite materials are much more effective for TET removal from aqueous solution than for BPA. This phenomenon is assigned to a difference in the TET/adsorbent interactions vs the BPA/adsorbent interactions: the decisive factor appears to be favorable electrostatic interactions for TET yielding a more effective TET removal.

## 1 Introduction

Water is a key resource for life, but industrialization, growing world population, accelerating urbanization, and climate change critically affect the availability of fresh water and predictions foresee a further decline in water availability and quality for the nearest future.^1^ For example, the World Health Organization (WHO) states that currently over “2 billion people live in water-stressed countries” and that this number will likely increase rather quickly.^2^

One of the central issues is that large fractions of surface waters are heavily contaminated.^3^ While there are other critical contaminants,^4^ increasing attention has been devoted to endocrine-disrupting chemicals (EDCs), a subgroup of the contaminants of emerging concern (CECs).^5^

Many EDCs are complex organic compounds that have become a global risk in the environment, especially in water systems. This is due to their high hydrophobicity, toxicity, and resistance to degradation. Generally speaking, EDCs show a high stability against light, heat, and oxidation, all of which leads to a recalcitrant and persistent nature.^6,7^ Previous studies have found more than 30 different CECs in untreated wastewater, treated wastewater, urban rainwater, agricultural rainwater, and fresh water.^8^ EDCs that have been found include, among many others, pharmaceuticals and bisphenols.^9−13^ Moreover, the COVID-19 pandemic has led to an increased use of personal care products and pharmaceuticals, which has caused additional environmental challenges as a result of wastewater discharged from hospitals and quarantine centers.^14^

**Figure 1:**
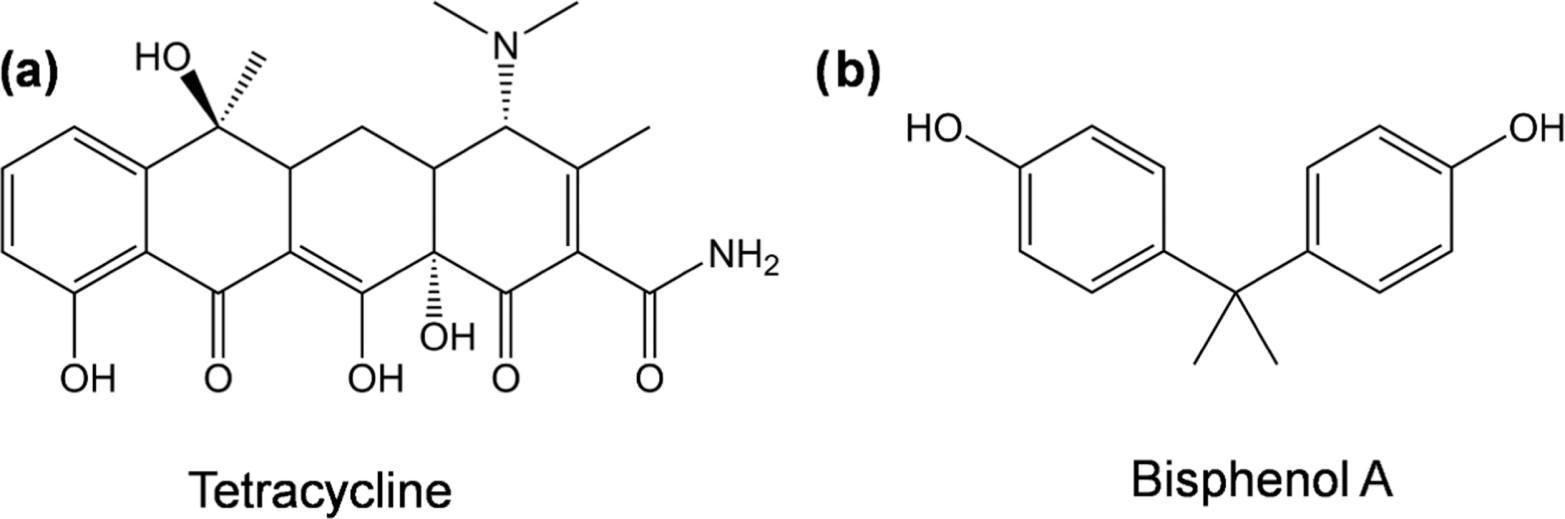
Chemical structure of (a) tetracycline (TET) and (b) bisphenol A (BPA).

Among the pharmaceuticals in use today, antibiotics are highly important. Among those, tetracycline (TET, Figure 1a) is most widely used to combat bacterial infections.^15^ It is frequently detected in local wastewater treatment plants because it is excreted from human bodies due to its low metabolism in the digestive system.^16^ TET concentrations range from ca. 1 μg L^–1^ in domestic wastewater to 20 μg L^–1^ in surface water, and ca. 100 μg L^–1^ in hospital wastewater.^17^

Besides pharmaceuticals, bisphenol A (BPA, Figure 1b), an industrial chemical used in consumer products like food packaging, dental sealants, plastic bottles, and baby feeding bottles,^18^ is another prime concern due to its severe toxicological and adverse health effects.^1,18−20^ BPA has been detected in all kinds of environmental water^19,21^ and BPA concentrations range from 0.1 mg/L in drinking water^22^ to 17.2 mg/L in hazardous waste landfill leachates.^22^

As a result, the presence of pollutants like BPA and TET in essentially all water bodies requires the development of strategies that are cheap, effective, and sustainable to provide access to safe water resources for everyone, especially the population in developing countries. Owing to the recalcitrant nature of these pollutants, however, the current water and wastewater treatment plants cannot handle their remediation^13^ and alternative strategies for water treatment are highly sought after. Indeed, several methods have been proposed for their removal such as biological treatment,^3,23^ membrane separation,^24^ photocatalytic degradation,^15,25−28^ adsorption,^29−31^ and chemical reduction.^32^

Adsorption is an established low cost, low tech but highly effective method for the removal of a wide range of contaminants.^33^ Especially porous and high-surface-area carbonaceous materials, such as graphene,^34−36^ activated carbon (AC),^4,37^ or carbon nanotubes,^38^ are popular adsorbents.

Recently, the application of titania (TiO_2_) nanoparticles (NPs) has attracted remarkable attention in several fields like biotechnology,^39−41^ air treatment,^41,42^ water splitting for hydrogen production,^43,44^ food and cosmetics,^45,46^ and wastewater treatment.^47,48^ Titania is inexpensive, chemically stable, nontoxic, and moderately hydrophobic under most conditions.^48^ Most previous studies have primarily focused on the photocatalytic properties of TiO_2_ NPs, while research into TiO_2_ adsorption properties is comparatively rare. Actually, TiO_2_ is an ideal adsorbent because its insolubility and point of zero charge (pH_pzc_) at neutral pH allow researchers to study its adsorption capacity over a wide range of pH.^49^ However, practical application of TiO_2_ NPs as an adsorbent remains limited due to its high aggregation tendency and due to challenges in separating the fine titania particles from treated water. This is an issue, as free TiO_2_ NPs left in the treated water are harmful as well.^50^

To overcome this problem, TiO_2_ NPs have been immobilized on various supports such as metal organic frameworks, clays,^51,52^ reduced graphene oxides,^53^ AC,^54,55^ and biochar^25,56^ which eliminates the need for time-consuming and expensive recovery processes. This approach improves both the adsorption properties and recovery efficiency of the resulting TiO_2_ NPs for water treatment applications. To the best of our knowledge, however, hybrid clay–TiO_2_ composites prepared via biomass-assisted synthetic routes for adsorptive removal of water contaminants have not been investigated so far.

Previous studies have, however, shown that agricultural biomass is a low-cost, readily available organic precursor of carbonaceous species, which possesses rich porosity (micropores and mesopores), abundant surface functional groups, and good stability.^25,27^ Also, the creation of high-surface-area nanoparticles and nano- to microarchitectures is facilitated by biomass degradation during calcination.^57^ In combination with a clay component, the materials become effective and easy to handle while remaining quite cheap in fabrication.

Therefore, the current study focuses on the synthesis and application of novel porous composites combining the advantages of orange-peel-based biochar, kaolin clay, and TiO_2_ into a new type of hybrid clay for the efficient removal of TET and BPA from aqueous solution.

## 2 Experimental Section

### 2.1. Materials and Reagents

Titanium tetraisopropoxide (98%), tetracycline (98%), bisphenol A (98%, all Sigma-Aldrich), and ethanol (99.8%, Carl Roth) were used as received. Raw kaolin clay was obtained from Redemption City, Mowe (6°48′0″N, 3°26′0″E.), Ogun State, Nigeria. Orange peel (OP) was sourced from the local REWE supermarket in Potsdam-Golm, Germany.

### 2.2. Carbonization of OP

OPs were washed with d.i. water, dried in an oven at 60 °C for 72 h, and subsequently milled until a fine powder was obtained. The powder was sieved with 250 μm mesh size to remove larger particles, and subsequently pyrolyzed in a furnace under argon between 300 and 600 °C for 2 h at a heating rate of 5 °C/min using a previously described setup.^58^ Upon cooling to room temperature, the material was removed from the oven, washed with distilled water, dried at 80 °C until constant weight was reached, and then stored in a tight container. Materials are labeled Cxxx (xxx = 300, 400, 500, 600), where C is for carbon and xxx indicates the calcination temperature.

### 2.3. Preparation of TiO_2_ Hybrid Clay Composites

For composite synthesis, 1.0 g of Cxxx and 1.0 g of kaolin clay were ground manually until macroscopically homogeneous. Then the powder was dispersed in 30 mL of anhydrous ethanol and the mixture was sonicated for 15 min. Then, 5 mL of titanium tetraisopropoxide were added to the mixture, followed by vigorous stirring for 1 h. Subsequently, 70 mL of deionized water was added dropwise under continued agitation over another hour. The resulting slurry was aged overnight, dried in an oven at 80 °C for 24 h, and then calcined in a furnace for 2 h at 500 °C under argon at a heating rate of 5 °C/min using a setup described earlier.^58^ After cooling to room temperature, the powder was washed neutral with d.i. water, dried to constant weight, and stored for future use. The products obtained are denoted C300KT, C400KT, C500KT, and C600KT (the numbers again indicate the calcination temperature, K is kaolin, T is TiO_2_). A neat TiO_2_/kaolin composite (denoted KT) and pure TiO_2_ powder (denoted P-TiO_2_) were also synthesized following the procedure described above but leaving out the respective components to produce a series of control materials.

### 2.4. Characterization

*The point of zero charge*, pH_pzc_, was determined via the salt addition method providing the net surface charge of the nanocomposite in suspension.^59^

*Powder X-ray diffraction* (PXRD) was done on a PANalytical Empyrean Powder X-ray diffractometer (Malvern, U.K.) in a Bragg–Brentano geometry equipped with a PIXcel1D detector using Cu Kα radiation (λ = 1.5419 Å) operating at 40 kV and 40 mA; θ/θ scans were run from 4 to 70° 2θ with a step size of 0.0131°, and a sample rotation time of 1 s.

*Scanning electron microscopy* imaging was done on a JEOL JSM-6510 SEM (Freising, Germany) equipped with an Oxford (INCAx-act SN detector) EDX detector.

*Elemental analysis* was done on an Elementar Vario EL III (Langenselbold, Germany) in duplicate.

*Attenuated total reflectance Fourier transform infrared (ATR-FTIR) spectroscopy* was done on a Nicolet iS5 (Thermo Scientific, Waltham, MA, iD7 ATR unit with a diamond crystal, resolution of 4 cm^–1^, 32 scans, from 400 to 4000 cm^–1^).

*Nitrogen sorption* measurements were done on a Micromeritics TriStar unit (Norcross, GA) at 77 K. Before the experiment, each sample was degassed to about 2 Pa at 353 K for 10 h. The specific surface area was calculated using the Brunauer–Emmett–Teller model.^60^ The average pore sizes were estimated from the adsorption branch of the isotherm using the Barrett–Joyner–Halenda method (BJH). The pore volume was determined at *P*/*P*_0_ > 0.99.^60^

*Thermogravimetric analysis* (TGA) was done on a Linseis TGA/DTA L81 (Selb, Germany) from 25 to 1000 °C under nitrogen with a heating rate of 10 °C/min.

### 2.5. Adsorption Experiments

Adsorption of TET and BPA on the composites was studied in batch adsorption mode and was conducted in duplicate. A 200 mg/L stock solution of each contaminant was prepared separately, from which several 20.0 mL aliquots of 20 mg/L TET and 5 mg/L BPA were prepared. All batch adsorption experiments were carried out in amber screw cap bottles covered with aluminum foil at room temperature.

First, a preliminary kinetic study was carried out to choose the composite material with the best adsorption performance. In each of these screening experiments, 20 mg of the composite was introduced into the solution containing either TET or BPA and agitated with an orbital shaker (IKA KS 260 basic) for 120 min at 150 rpm at room temperature. Then the suspension was filtered through PTFE syringe filters (VWR, 0.45 μm) and the concentrations of TET and BPA were quantified with UV–vis spectroscopy at a wavelength of 358 and 225 nm (the absorption maxima of the respective contaminants), respectively.

The adsorption capacity and percentage removal of the pollutant were calculated using eqs 1 and 2, respectively
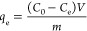
1
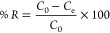
2where *q*_e_ is the amount of pollutant adsorbed (mg/g); *C*_0_ and *C*_e_ are the initial and equilibrium liquid-phase concentrations after a set time (mg/L), respectively; *V* is the volume of the solution (L); *m* is the mass of the composite used (g); and %*R* is percent pollutant removal.

Furthermore, the influence of some process variables on the sorption efficiency using the most efficient nanocomposite (C300KT) was studied in detail. The effect of the

(1) adsorbent mass (10, 20, 30, 40, 50, and 60 mg) at a fixed pollutant concentration,
(2) initial pollutant concentration (5–80 mg/L) at a fixed dose of adsorbent (20 mg),
(3) solution pH (3.0, 5.0, 7.0, 9.0, and 11.0) where the pH was adjusted with 0.1 M HCl and 0.1 M NaOH,
(4) initial ionic strength of the pollutant solution (0.001, 0.01, and 0.1 mol/L NaCl), and
(5) foreign ions (Cl^–^, HCO_3_^-^, SO_4_^2-^, Cu^2+^, Na^+^, and K^+^) at a fixed concentration were studied.

All experiments were run up to 120 min. The equilibrium data obtained were analyzed with Langmuir, Freundlich, Temkin, Brouers–Sotolongo (B.S.), and Langmuir–Freundlich models. Kinetic data were analyzed with pseudo-first-order, pseudo-second-order, Brouers–Sotolongo fractal (B.S.F.), and Elovich kinetic models. All relevant equations are given in the Supplementary Information (S.I., Tables S1 and S2).

## 3 Results and Discussion

**Figure 2:**
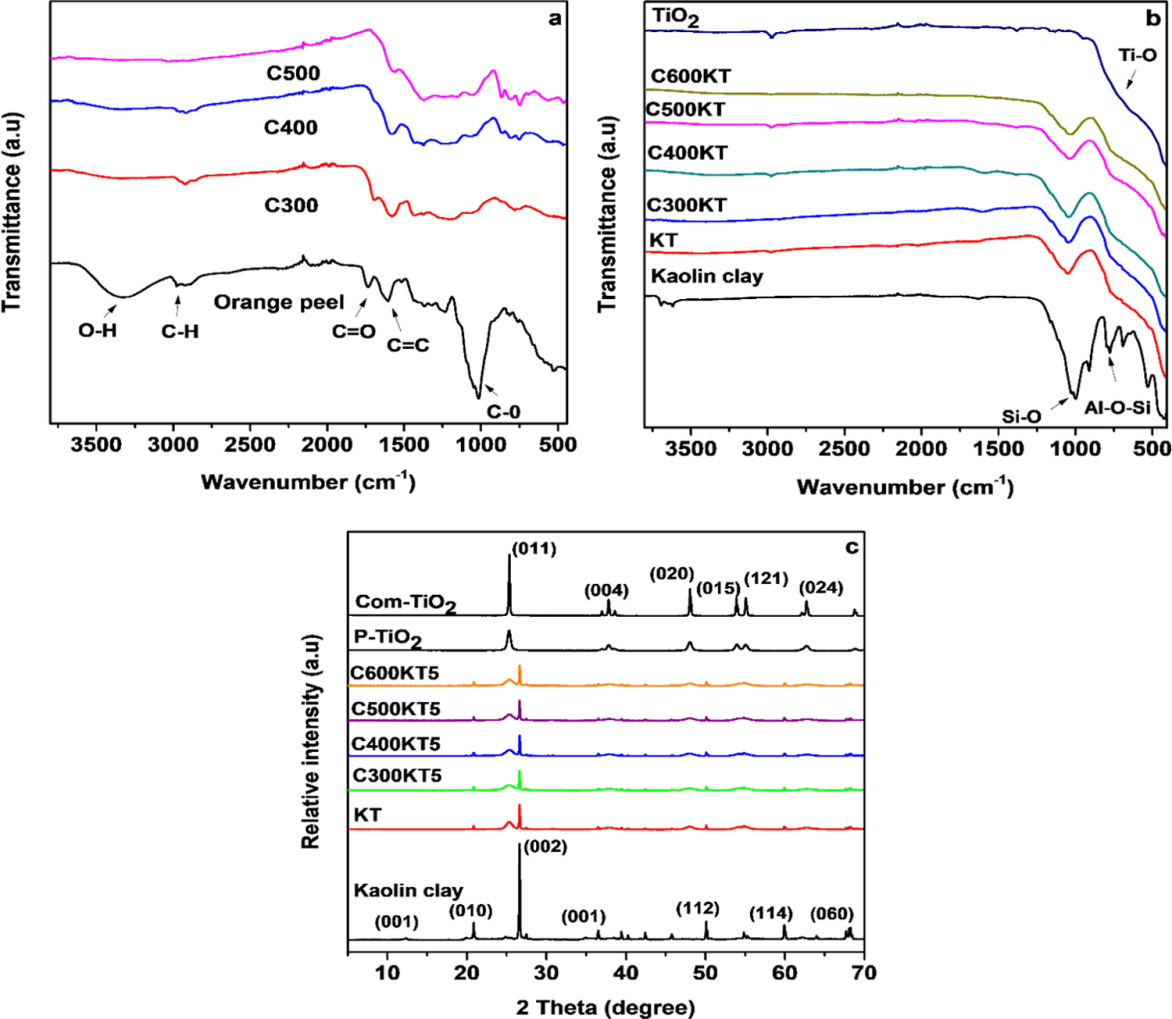
(a) ATR-FTIR spectra of raw OP and Cxxx biochars, (b) ATR-FTIR spectra of kaolin and the TiO_2_ composites CxxxKT, and (c) XRD patterns obtained from kaolin, TiO_2_, and the TiO_2_ composites. Com-TiO_2_ is commercial titania used for comparison.

The ATR-FTIR spectrum of the raw OP and the spectra of the biochar obtained from OP calcination at different temperatures are shown in Figure 2a. In the spectrum of the raw OP, a medium band observed at 3578 to 3027 cm^–1^ is assigned to the O–H stretch of lignin.^61,62^ The band at 2923 cm^–1^ is assigned to a C–H stretch vibration, and the sharp bands observed at 1746 and 1613 cm^–1^ are attributed to a C=O stretch vibration of carboxylic acids or esters and to a C=C stretch vibration of aliphatic and aromatic moieties, respectively.^63^ The intense band at 1016 cm^–1^ is a C–O stretch vibration of −OH or −C–O–C– in ester groups.^64^ Also, the overlapping bands from 1442 to 1182 cm^–1^ may be attributed to signals from −CH_2_– and −CH_3_– vibrations of aliphatic chains in the lignocellulose and to not fully resolved C–H bend and C–N stretch vibrations.^65^

After pyrolysis of the OP, the IR spectra of the products C300 to C600 show a strongly reduced signal intensity. The remaining bands can again be assigned to C–H, C–O, C=O, and C=C bonds, as displayed in Figure 2a. As an exception, the IR spectrum of C600 (not shown) shows no distinctive features. This could be due to the presence of graphenelike species in C600.^66^

The IR spectra of the final carbon/kaolin clay/TiO_2_ composites CxxxKT are shown in Figure 2b. The band at 1018 cm^–1^ is attributed to the Si–O bending vibration of kaolin and quartz. The broad signal between 600 and 700 cm^–1^ is assigned to a Ti–O–Ti stretching vibration.^67,68^ Moreover, the heat treatment also leads to a significant loss of functional groups in the biochar as is shown by reduced intensities of some IR bands.^69^ It must be noted, however, that generally the spectra are very poor with very broad signals and that all IR signal assignments are to be treated with care. In spite of this, the general observation of a loss of functional groups is confirmed by elemental analysis, Table 1 below.

**Table 1:** Elemental Analysis and Nitrogen Sorption Data of the Biochars, the Control Material P-TiO_2_, and the Final Composites^a^

material	C300	C400	C500	C600	P-TiO_2_	C300KT	C400KT	C500KT	C600KT
C (At %)	68.80	75.65	77.63	79.30	0.28	19.70	22.39	22.93	23.99
H (At %)	4.88	4.17	2.73	1.73	0.056	0.89	0.92	0.86	0.60
N (At %)	1.83	1.73	1.64	1.39	0.039	0.59	0.53	0.51	0.62
*S*_BET_ (m^2^/g)					55.22	150.87	163.20	84.25	70.97
pore volume (cm^3^/g)					0.12	0.15	0.18	0.16	0.15
pore diameter (nm)					10.63	6.34	7.43	9.95	9.58

a Surface area in C300, C400, C500, and C600 is too low for useful data; surface area < ca. 10 m^2^/g, and At % represents atomic weight percent.

Figure 2c shows representative XRD patterns obtained from the composites. All indexed reflections, except for the (001) stemming from the kaolin clay, can be assigned to the presence of quartz. In all patterns, the two main quartz reflections are clearly visible at 20.8 and 26.6°. Additionally, the typical diffraction peaks of anatase TiO_2_ (ICSD 154603) are observed in the XRD patterns of P-TiO_2_. The reflections are somewhat broader than in a reference commercial TiO_2_ indicating that the TiO_2_ components in the composites are in fact nanocrystalline with a crystallite size of around 60 nm for all composites based on the Scherrer equation^70^ from the only superposition-free (020) reflection at 47.93° 2θ.

**Figure 3:**
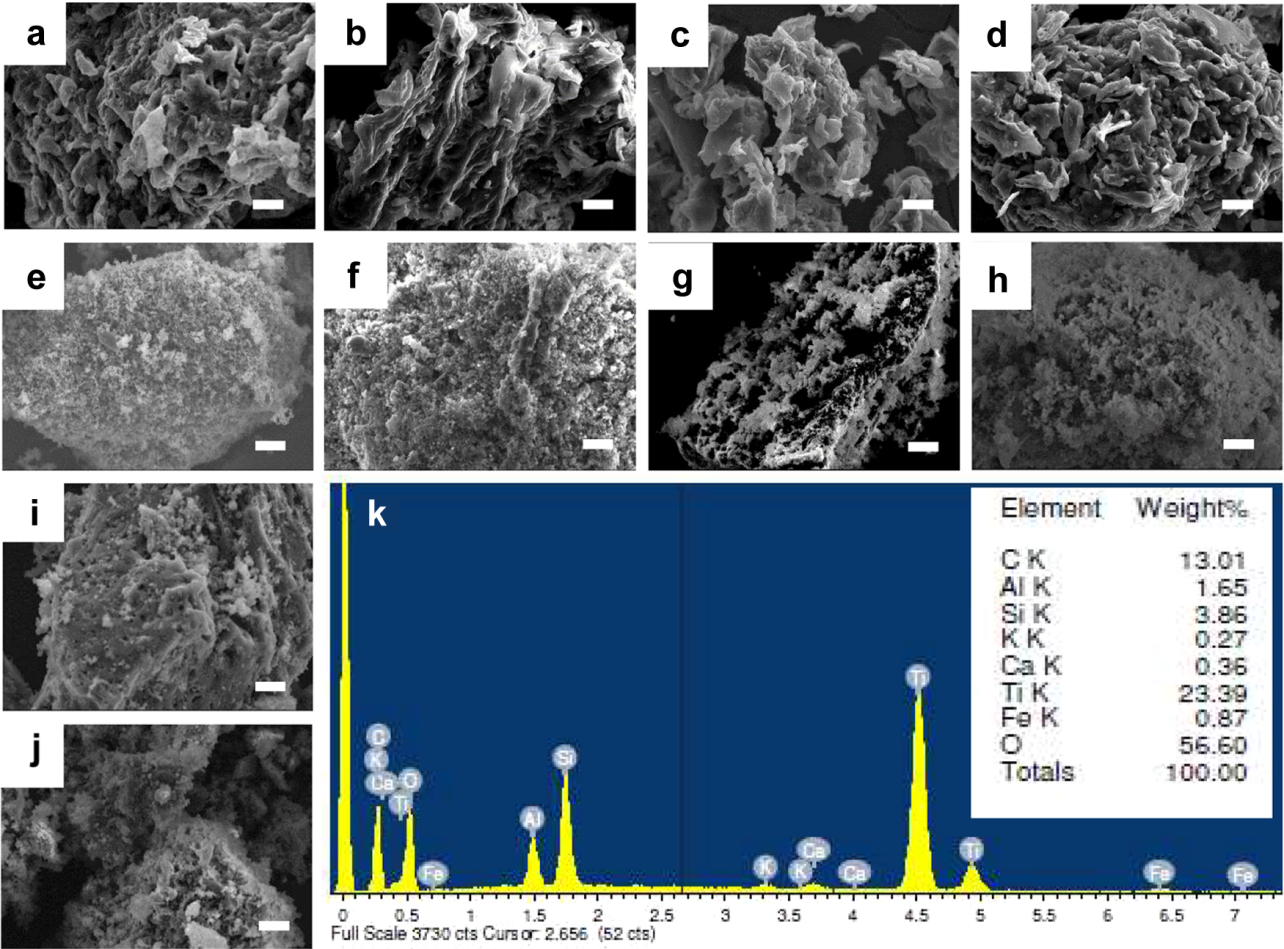
SEM images of (a) C300, (b) C400, (c) C500, (d) C600, (e) P-TiO_2_, (f) KT, (g) C300KT, (h) C400KT, (i) C500KT, (j) C600KT, and (k) EDX spectrum of C300KT. Scale bar is 10 μm in all images.

Figure 3 shows scanning electron microscopy images of the materials. The biochars (Figure 3a–d) exhibit a flowerlike heterogeneous porous structure, with an irregular intercellular spacing. A similar observation was reported by Mafra and co-workers.^71^ Essentially all biochars C300 to C600 are very similar and show no significant morphological differences.

P-TiO_2_ (Figure 3e) contains densely agglomerated particles, while the KT and TiO_2_ hybrid clay composites show well-dispersed nanometer-sized particles (presumably TiO_2_) deposited on the carbon/kaolin clay support, Figure 3f–j. Energy dispersive X-ray (EDX) spectroscopy of a select example, C300KT (Figure 3k–l), detects Ti and O indicative of TiO_2_ and further elements such as C, Al, Si, K, Ca, and Fe indicating the presence of the kaolin clay.

Table 1 shows the elemental composition of the composites. First, elemental analysis (EA) shows that the carbon content increases with increasing calcination temperature. At the same time, H and N contents decrease with increasing calcination temperature. These values therefore indicate a continuous transformation to high-carbon-content materials, consistent with IR data (Figure 2 above). As the synthesis of the composites starting from the biochar was always done at 500 °C, the final C content is between ca. 20 and ca. 24% without larger variation. For H and N, there is no clear trend in terms of the content in the composites.

Table 1 also summarizes the data obtained from the nitrogen sorption experiments. All measurements show a distinctive type IV isotherm (S.I., Figure S1), which is typical of mesoporous materials. A small fraction of micropores are observed in all composites. P-TiO_2_ has the lowest surface area (55 m^2^/g) while the surface area of the hybrid clay impregnated with TiO_2_ is higher (S.I., Figure S1).

Somewhat surprisingly, a decrease in the surface area is observed from C400KT to C600KT (151–71 m^2^/g), that is, the surface area decreases with increasing pyrolysis temperature. Possibly, this is caused by a densification or agglomeration of the TiO_2_ particles around the pore entrances or the clogging of some pores by the growth of titania NPs. The concurrent increase in pore diameter from C300KT to C600KT reveals, however, that there is no obvious blocking of the mesopores and that the mesoporous nature of the materials is retained. More likely, therefore, the reduction of the surface area could be due to the onset of graphitization in the carbonaceous components and a concurrent densification of the entire material.

**Figure 4:**
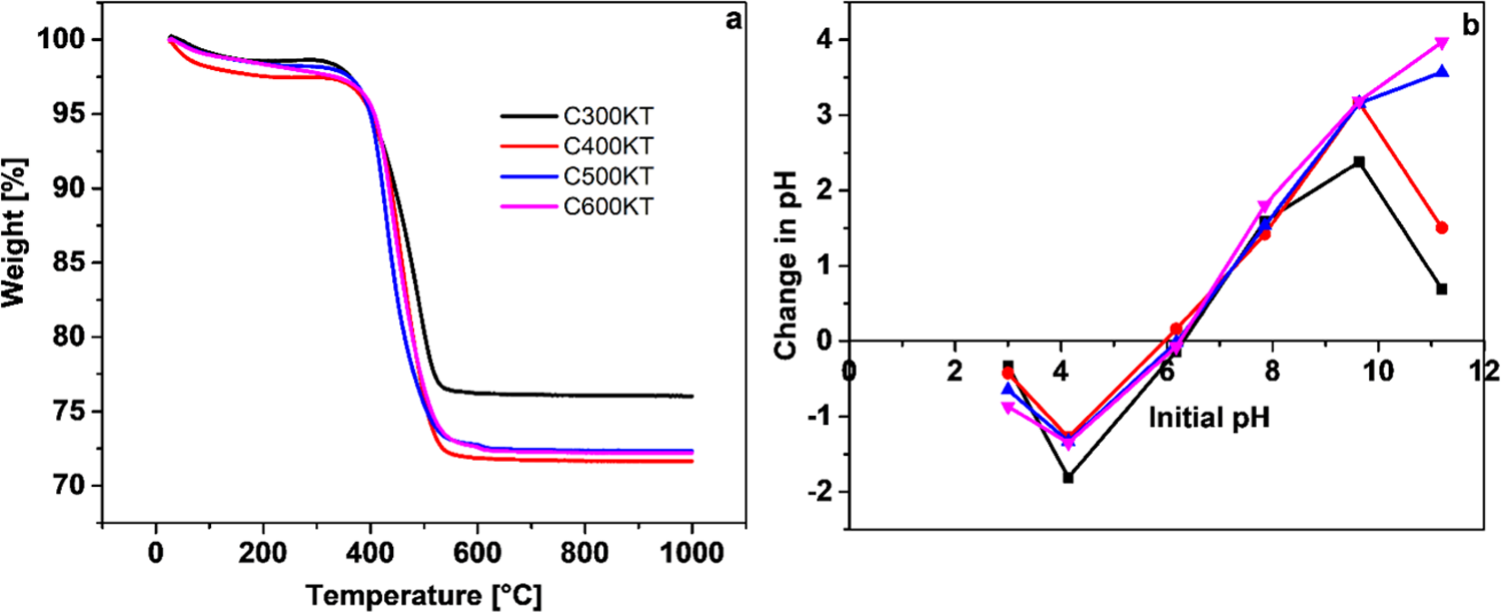
(a) TGA data and (b) pH_pzc_ plots obtained for C300KT to C600KT.

Figure 4a shows thermogravimetric analysis (TGA) data. A first weight loss is observed between 25 and 115 °C for all nanocomposites. This is attributed to the evaporation of physisorbed water and other solvents along with condensation processes releasing water upon heating.^25,72^ A further weight loss of ca. 22% is observed between 420 and ca. 538 °C. These weight losses are likely due to the decomposition of carbonaceous material from the biomass precursors along with further solvent evaporation from condensation processes in the inorganic components.

Generally, C400KT, C500KT, and C600KT show very similar TGA data, but the TGA data of C300KT show a much higher residual mass at higher temperatures. This is likely because the starting material C300 has a somewhat lower carbon fraction than the other three starting materials.

Figure 4b shows the point of zero charge, pH_pzc_. The pH_pzc_ allows for the determination of the net surface charge and the pH at which the particles are essentially neutral.^59^ The information provided by the pH_pzc_ can be used to adjust the surface charge for optimized removal of a particular pollutant from solution by altering the net charge of the adsorbents. The pH_pzc_ is 6.30, 5.96, 6.14, and 6.25 for C300KT, C400KT, C500KT, and C600KT, respectively. These data indicate that the composite particles are positively charged at pH < pH_pzc_ and the particles are negatively charged at pH > pH_pzc_.

In the remainder of the study, we will concentrate on one material, C300KT, because this composite has shown the best adsorption capability in the preliminary tests (S.I., Figure S2), that is, C300KT has shown the highest removal rates among all composites studied.

**Figure 5:**
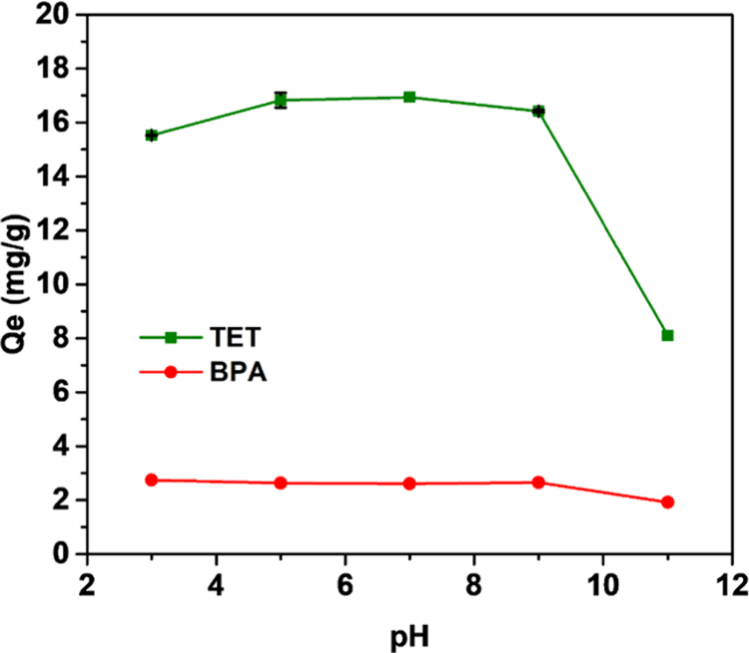
Effect of pH on BPA and TET adsorption on C300KT. Note that the error bars in the data for BPA adsorption are on the order of the red symbols indicating a very high reproducibility of the experiments.

The pH of the contact solution is an important influence on an adsorbent’s surface charge, the dissociation of functional groups, the ionization degree, and charge of some pollutants.^30^Figure 5 shows the effect of pH on the adsorption of TET and BPA onto C300KT. For BPA, the pH does not seem to be a strong influence, as throughout the observed pH range, no significant change was observed. This suggests that surface-charge-dependent electrostatic forces are not a key parameter for adsorption of BPA onto C300KT. Possibly, this is because BPA is a neutral molecule and therefore does not respond strongly to surface charges. It must be noted, however, that the reproducibility of this experiment is extremely high and that the error in these experiments is smaller than the symbols in the figure. As a result, the slight decrease in the adsorption efficiency at high pH indicates that some small change may change the interaction between BPA and C300KT. The details of this interaction are, however, unresolved to date.

In contrast, for TET, a sharp decrease in the adsorption capacity is observed at pH > 9, Figure 5. This is a result of electrostatic repulsion between C300KT and the TET molecules at higher pH. In an alkaline environment, the anionic species of TET predominate due to the deprotonation of the tricarbonyl as well as the phenolic diketone moiety^17^ while the surface of C300KT is already negative at pH > 6.2 (pH_pzc_ = 6.2). A very weak π–π interaction on top of the charge–charge repulsion may not be able to overcome this unfavorable interaction at pH > 9.^17^ As a result, a solution pH below ca. 6.2 is much more suitable for TET adsorption on C300KT. Similar to above, the reproducibility of these measurements is very high and the best pH range for TET removal is pH 6–7.

**Figure 6:**
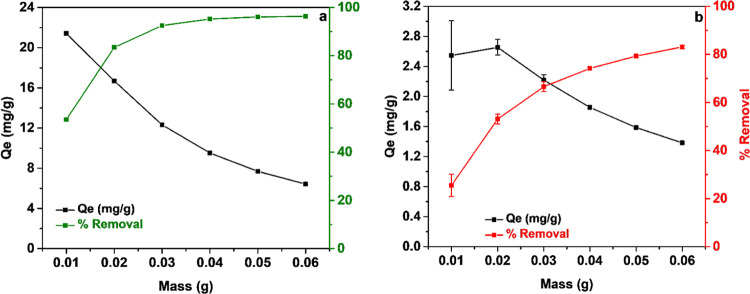
Effect of adsorbent mass on (a) TET and (b) BPA adsorption on C300KT. Again the reproducibility of the experiments shown in panel (a) is high and error bars are too small to see.

Besides pH, the effect of the adsorbent mass is important for real-life applications. Figure 6 shows that increasing adsorbent mass leads to a decrease in adsorption capacity from 21.42 to 6.42 mg/g for TET as the added mass of C300KT increases. The same trend is observed for BPA where the adsorption capacity declines from 2.46 to 1.38 mg/g. However, if considering the fraction of removed pollutant, the addition of more adsorbent leads to more pollutant removed. For TET, the removal rate increases from 54 to 96% upon increasing the adsorbent concentrations, and for BPA, the removal rate improves from 25 to 83%. Likely, this increased removal rate is a result of increased surface area and higher number of active sites provided by the adsorbent.

**Figure 7:**
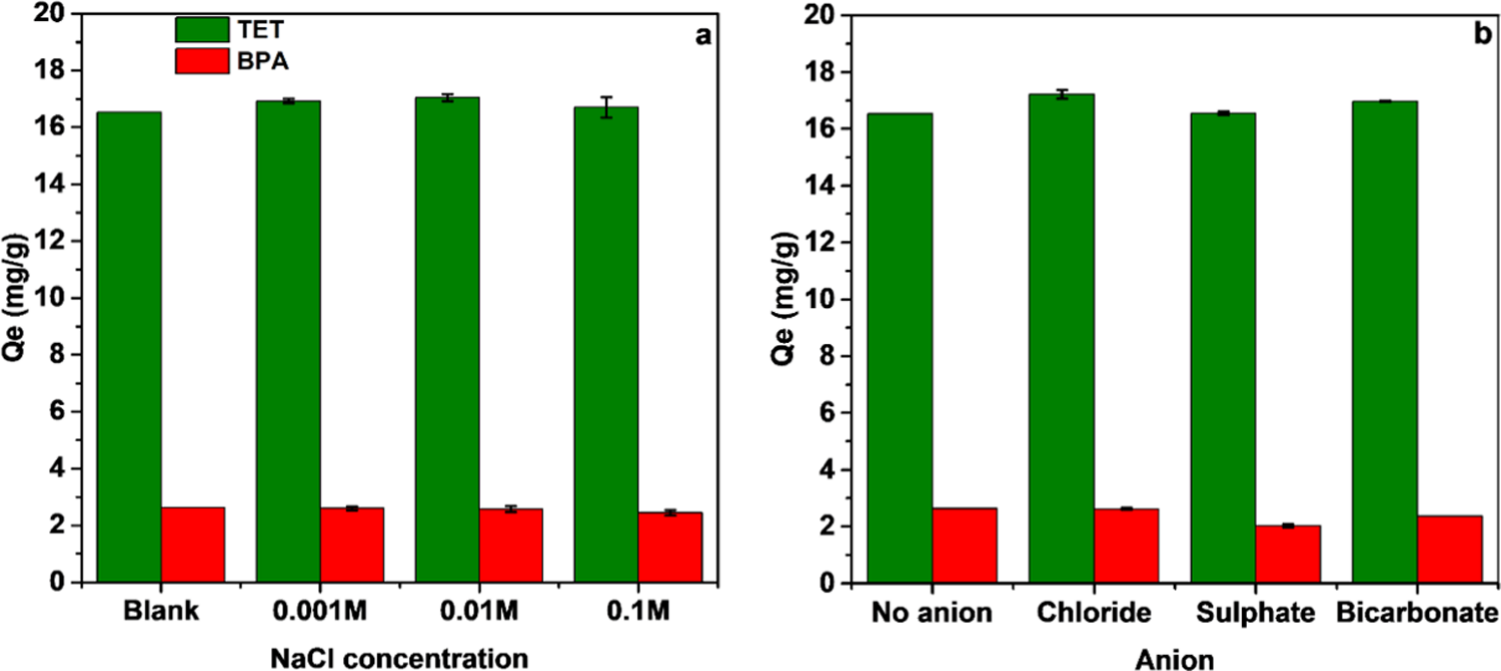
Effect of ionic strength (a) and anion type (b) on the adsorption of TET and BPA on C300KT.

Besides pH and adsorbent mass, the ionic strength of a solution is a key parameter to control pollutant adsorption on a given adsorbent.^73^ Generally, industrial effluents contain a high amount of salts; this directly affects the ionic strength of these waters and can influence removal efficiency of the pollutants.^74^Figure 7a shows, however, that the adsorption capacities of the current composite, C300KT, are only very weakly affected by the ionic strength, as exemplified by NaCl solutions with different concentrations. In essence, these data show that chloride ions do not compete with the TET and BPA during the adsorption process across a wide range of ionic strengths.

Anions are pervasive in most wastewaters and concentrations of about 1 mM of anions like HCO_3_^–^ or SO_4_^2–^ have been found in surface water.^75^ Therefore, not only the effect of ion concentration but also the effect of the ion type, especially the anion type, is important to evaluate the effectiveness of an adsorbent. Figure 7b shows that there is a slight increase in the adsorption capacity of C300KT for TET adsorption in the presence of Cl^-^ and HCO_3_^–^. This increase may be attributed to the salting-out effect.^76^ In contrast, SO_4_^2–^ has no significant effect on TET adsorption on C300KT, but slightly reduces the BPA adsorption on C300KT. This may be a result of competition between the sulfate ion and BPA species for the available active sites on C300KT, but the exact mechanism of this competition is not clear.

**Figure 8:**
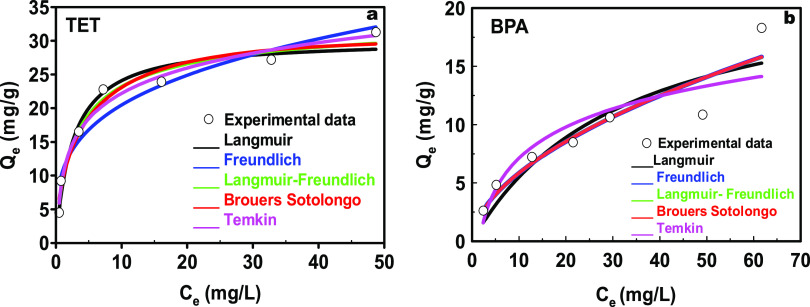
Adsorption isotherms for the adsorption of (a) TET and (b) BPA on C300KT.

Figure 8 shows the adsorption isotherms for both adsorbent/adsorbate pairs, C300KT/BPA and C300KT/TET, to characterize the adsorbent/adsorbate interaction and the distribution of pollutant molecules between the liquid and solid phase at equilibrium.^77^ The experimental data were fitted to five nonlinear theoretical models, Figure 8. The exact parameters extracted from fitting with the different isotherms are presented in Table S1 (S.I.).

The Langmuir model assumes that all sorption sites are identical and the adsorbate is distributed as a monolayer over a homogeneous surface.^78^ According to the values obtained from the fitting (Table S1, S.I.), the Langmuir model shows correlation coefficients (*R*^2^) of 0.982 and 0.892 for TET and BPA, respectively. The Langmuir maximum adsorption capacity, *q*_m_, for TET and BPA is 30.25 and 23.27 mg/g, respectively, which are comparable to similar data, Tables S3 and S4 (S.I).

The separation factor is also an important parameter used to assess whether an adsorption process is favorable (0 < *R*_L_ < 1), unfavorable (*R*_L_ > 1), or irreversible (*R*_L_ = 0).^77^ The *R*_L_ values for TET and BPA are 0.391 and 0.031, respectively, indicating that the adsorption of TET and BPA onto C300KT in the studied concentration range is favorable.

Based on the highest *R*^2^ value obtained, the Temkin (*R*^2^ = 0.987) and Freundlich (*R*^2^ = 0.925) models best fit the sorption data for TET and BPA, respectively. The Temkin model assumes that the heat of TET adsorption decreases linearly with the coverage of C300KT due to interactions. The Temkin constant, *b*_T_, is defined as a variation of adsorption energy, which indicates that the adsorption process is endothermic (*b*_T_ < 1) or exothermic (*b*_T_ > 1).^79^ The *b*_T_ value for TET adsorption is 5.42 suggesting an exothermic adsorption. This in turn indicates that there is an electrostatic interaction taking place and the heterogeneity of C300KT pore surface plays an important role in TET adsorption.^80^

The Freundlich model is based on the phenomena that adsorption of BPA occurs on a heterogeneous surface with several mechanisms involved, where *n* is a parameter known as heterogeneity factor. The *n* value can be used to evaluate when the adsorption process is linear (*n* = 1), physical (*n* > 1), or chemical (*n* < 1). In the current study, *n* obtained for BPA adsorption is 1.831 suggesting that the major interaction is physisorption. Indeed, physisorption is consistent with the data obtained for the effects of ionic strength (Figure 7a), which then suggests that a major fraction of the interaction is based on van der Waals forces.

**Figure 9:**
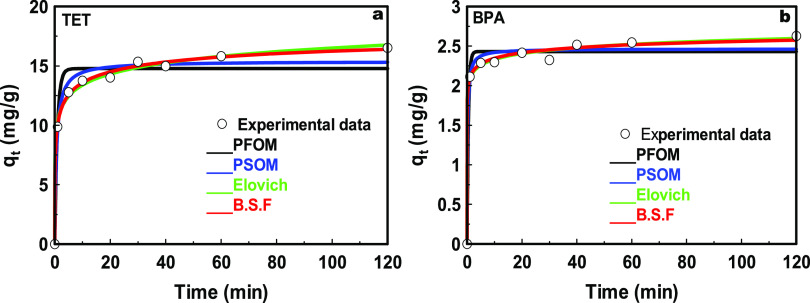
Kinetic model fits for the adsorption of (a) TET and (b) BPA on C300KT.

Figure 9 shows the fits of the kinetic data obtained from the adsorption of TET and BPA on C300KT to several kinetic models: Elovich, B.S.F., PFOM, and PSOM. The kinetic parameters obtained for all fitting models are presented in Table S2 (S.I.). Generally speaking, the analysis of the kinetic data shows that *q*_e,cal_ (calculated adsorption capacity) and *q*_e,exp_ (experimental adsorption capacity) are quite close for both TET and BPA. Based on *R*^2^ and the sum of the squared errors^75^ obtained for the different fits, the model fits can be ranked as B.S.F. > Elovich > PSOM > PFOM for TET and Elovich > B.S.F. > PSOM > PFOM for BPA. As a result, a few conclusions can be drawn as follows:

(1) The Elovich model is based on chemisorption through bond sharing and interactions. As the fits with the Elovich model are highly ranked, the data suggest that the adsorption of both TET and BPA involves chemical interactions. These interactions are likely realized via the functional groups of the pollutants (−OH, −C=O, etc.) and the composite surface (−Ti–OH, −COOH, −C=O, −Si–O– from the kaolin, etc.).^81^
(2) From the adsorption isotherms (Figure 8), it can be inferred that C300KT has a heterogeneous surface and the fact that the B.S.F model is a good approach to fit the kinetic data implies that there are different adsorption sites on the heterogeneous surface of C300KT with different affinities for adsorption.^82^
(3) The B.S.F. model also implies that adsorption occurs via a complex mechanism involving various attractive forces such as π–π, π–cation, and van der Waals forces.^74^ While hard to prove in such a complex material, π–π interactions could be present between the aromatic moieties of the pollutants and some graphenelike sections on the adsorbent. This is however rather speculative at the moment, as there is no direct proof for the presence of aromatic or graphenelike sections from IR spectroscopy.

## 4 Adsorption Mechanism

In general, several interactions such as hydrogen bonding, electrostatic interactions, hydrophobic interactions, or van der Waals forces are involved in the adsorption of organic pollutants on different adsorbents.^83,84^ Primarily, the adsorption mechanism depends on operating conditions such as pH, temperature, or ionic strength which affect, for example, the surface charge or the degree of protonation of the adsorbent.

In the current case of TET removal with C300KT, electrostatic repulsion appears to be a dominating force at basic conditions (pH > 9) because both the adsorbent and the adsorbate are negatively charged. This indicates that for TET, while other forces such as van der Waals may be relevant as well, the entire process is mostly governed by electrostatics. This is consistent with the effects observed for changing ion strengths and ion types (Figure 7a) along with IR spectroscopic data (Figure 2b).

Unlike the case of TET, adsorption of BPA on C300KT appears to *not* be governed by electrostatics, as can be concluded from the pH dependence and the very weak effects of ionic strength on adsorption (Figure 7a). Likely this is because BPA is uncharged and other forces like hydrogen bonding or van der Waals become more prominent.

Finally, the kinetic data (S.I., Table S2) show that the Elovich and B.S.F. models fit the adsorption kinetics quite well, while the PFOM and PSOM models are much less accurate. This again suggests that there is a strong contribution from chemisorption for TET in addition to weaker interactions such as van der Waals forces in both cases.

## 5 Conclusions

The study describes a new low-cost adsorbent for tetracycline (TET) and bisphenol A (BPA) removal from aqueous solution and demonstrates evidence that the combination of biochar, titania, and kaolin clay provides access to effective yet cheap materials for water treatment. The synthesis is straightforward and the porous powders effectively remove both pollutants from solution. The material based on the biochar prepared at the lowest temperature (300 °C) is the most effective. Kinetic and equilibrium measurements indicate that the adsorption/removal process works through a combination of electrostatics and weaker forces for TET, while for BPA weaker forces such as van der Waals and hydrogen bonding appear to be the main interactions. Increasing dosage of the C300KT adsorbent increases the removal rate while generally TET is removed much more effectively than BPA. Overall, the new adsorbent C300KT is a cheap yet effective new adsorbent for removal of TET and BPA from aqueous solution.

## Supporting Information Available

N_2_ adsorption–desorption isotherms and corresponding BJH pore-size distribution curves of TiO_2_ and the composite materials (Figure S1); adsorption efficiencies of all composites for removal of 20 mg/L TET and 5 mg/L BPA (Figure S2); comparison between the separation ability of C300KT and unmodified TiO_2_ NPs in aqueous media after approx. an hour (Figure S3); isotherm model equations and parameters for the adsorption of TET and BPA onto C300KT composite (Table S1); nonlinear kinetic model equations and parameters for the adsorption of TET and BPA onto C300KT composite (Table S2); comparison of adsorption capacities for TET on C300KT with literature (Table S3); and comparison of adsorption capacities for BPA on C300KT with literature (Table S4) (PDF)
