# Supplementary material for: Efficient Removal of Tetracycline and Bisphenol A from Water with a New Hybrid Clay/TiO2 Composite
Source: ACS Omega. 2023 Jun 5;8(24):21594–604. doi: 10.1021/acsomega.3c00184 (PMC10286278; doi:10.1021/acsomega.3c00184)
Supplement: Supplementary file 1 — ao3c00184_si_001.pdf [file ao3c00184_si_001.pdf]

Efficient removal of tetracycline and bisphenol A from water with a new hybrid clay/TiO_2 composite

Morenike O. Adesina,[1,2,3,4,*] Inga Block,[1] Christina Günter,[5] Emmanuel I. Unuabonah,[2,3] and Andreas Taubert[1,*]

[1] Institute of Chemistry, University of Potsdam, D-14476 Potsdam, Germany.

[2] African Centre of Excellence for Water and Environment Research (ACEWATER), Redeemer's University, PMB 230, Ede, Osun State, Nigeria.

[3] Department of Chemical Sciences, Redeemer's University, PMB 230, Ede, Osun State, Nigeria.

[4] Lead City University, Ibadan, Oyo State, Nigeria.

[5] Institute of Geosciences, University of Potsdam, D-14476 Potsdam, Germany

* corresponding author: M.A. morenicky1@yahoo.com

* corresponding author: A.T. ataubert@uni-potsdam.de

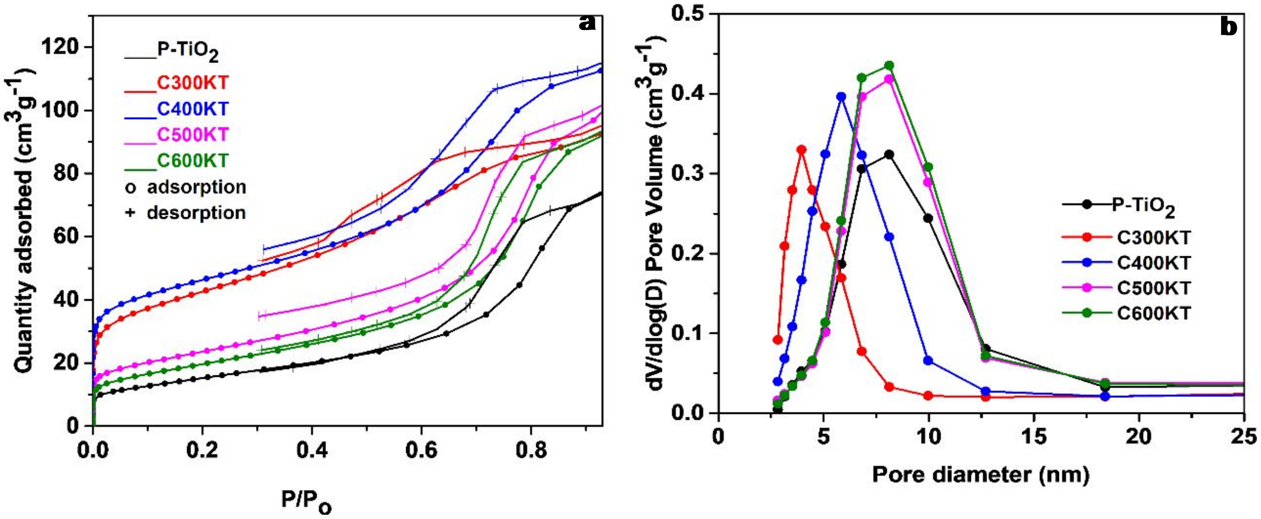

Figure S1 a) N_2 adsorption-desorption isotherms b) and corresponding BJH pore-size distribution curves of TiO_2 and the composite materials

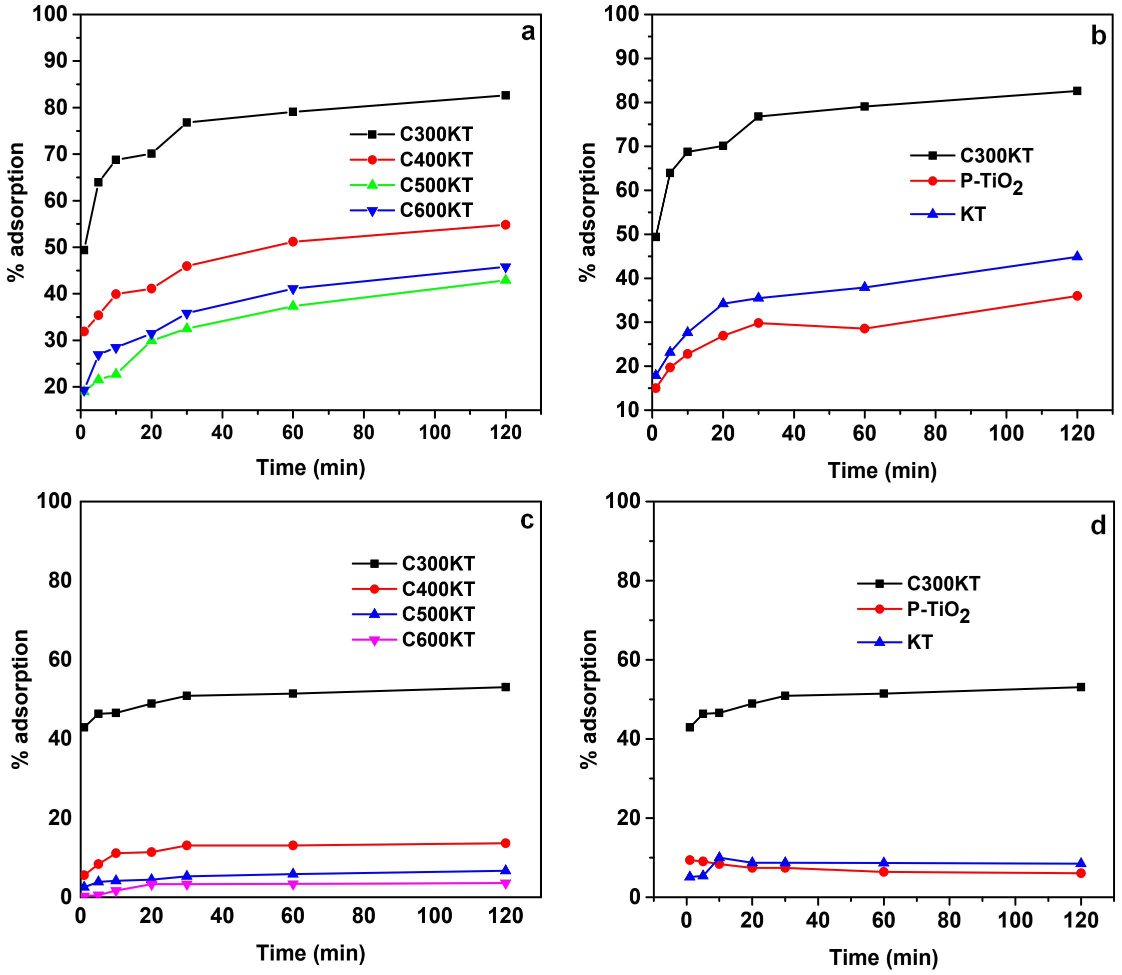

Figure S2: Comparative kinetic study; Adsorption efficiencies of all composites for removal of 20 mg/L TET a) and b), and 5 mg/L BPA (c) and (d) (conditions: adsorbent mass: 20 mg, volume of solution: 20 mL)

Table S1: Isotherm model equations and parameters for the adsorption of TET and BPA onto C300KT composite

Model	Equation	Parameters	TET	BPA
**Langmuir**	$q_e = \frac{q_m.K_L.C_e}{1 + K_L.Ce}$	q_m(mg/g)<br>K_L (L/mg)<br>R^2	30.250<br>0.391<br>0.982	23.266<br>0.031<br>0.892
**Freundlich**	$q_e = K_F.C_e{}^{1/n}$	K_F (mg/g)<br>n<br>R^2	10.655<br>3.529<br>0.954	1.664<br>1.831<br>0.925

**Temkin**	$q_e = \frac{RT}{b_T}\ln\left(K_T.C_e\right)$	b_T (J/mol)<br>k_T (L/g)<br>R^2	5.423<br>6.003<br>0.987	3.851<br>3.851<br>0.876
**Langmuir-Freundlich**	$q_e = \frac{q_m.(K_{LF}.C_e)^{1/n}}{1+\ (K_{LF}.C_e)^{1/n}}$	q_m(mg/g)<br>K_{LF}<br>n<br>R^2	33.461<br>0.286<br>0.774<br>0.985	471.414<br>3.83 e-5<br>0.5564<br>0.924
**Brouers-Sotolongo**	$q_e = q_{BS}(1 - \exp\left(-K_{BS}{C_e}^{\alpha_{BS}}\right))$	q_{bs} (mg/g)<br>K_{BS} (L/mg)<br>α_{BS}<br>R^2	30.294<br>0.367<br>0.593<br>0.981	488.773<br>0.004<br>0.5511<br>0.924

*** R^2= correlation coefficient**

q_e is the amount of solute adsorbed per unit weight of adsorbent (mg/g), C_e is the equilibrium concentration of solute in the bulk solution (mg/L), q_m is the maximum monolayer adsorption capacity (mg/g),K_L is the constant related to the affinity of the adsorbate to the sorbent, K_{LF} is Langmuir-Freundlich constant, A_T is the temkin equilibrium binding constant (L/mg) corresponding to maximum binding energy and B is the isotherm constant related to the heat of adsorption (J/mol), b is a constant, R is gas constant (8.314 J/mol/K) and T is temperature (K), q_{BS} is the saturation capacity in mg/g; K_{BS} is K_F/q_{BS} where K_F is the Freundlich constant; α_{BS} is a measure of the width of the sorption energy distribution, K_F is an empirical constant indicative of the relative adsorption capacity of the adsorbent and 1/n is an adsorption constant that shows the intensity of heterogeneity of adsorption.

Table S2: Non-linear kinetic model equations and parameters for the adsorption of TET and BPA onto C300KT composite

**Model**	**Equation**	**Parameters**	**TET**	**BPA**
Experimental data	See main article, eq. 2	$q_{e,exp}$ (mg/g)	16.520	2.62
Pseudo first-order	$q_t = q_e(1 - \exp\left(-K_1 t\right))$	K_1 (L min^{-1})<br>$q_{e,\,cal}$ (mg/g)<br>R^2<br>SSE	14.770<br>1.079<br>0.952<br>1.406	2.432<br>2.034<br>0.979<br>0.016

Pseudo second-order	$q_t = \frac{q_e^2 K_2 t}{q_e K_2 t + 1}$	K_2 (L min $^{-1}$)<br>$q_{e,cal}$ (mg/g)<br>R^2<br>SSE	0.099<br>15.396<br>0.979<br>0.600	2.142<br>2.466<br>0.985<br>0.011
Elovich	$q_t = \frac{1}{\beta}(\ln(1 + \alpha\beta t)$	B (gmg^{-1})<br>α ($mgg^{-1}min^{-1}$)<br>R^2<br>SSE	0.737<br>2616.030<br>0.996<br>0.123	9.345<br>31259<br>0.996<br>0.003
Brouers-Sotolongo Fractal kinetics	$q_{n,a}(t) = qe[1 - [1 + [n-1][t/\tau_{n33,a}]^{\alpha}]^{-1/(n-1)}$	qe (mg/g)<br>n<br>τ<br>α<br>R^2<br>SSE	19.041<br>4.212<br>64.635<br>0.327<br>0.997<br>0.118	3.085<br>1.137<br>2.11 e-6<br>0.138<br>0.995<br>0.005

*** SSE = Sum of squared errors , R^2= correlation coefficient**

q_e and q_t are the amounts adsorbed at equilibrium and time, t (mg/g), k_1 is the first order rate constant (g/mg.min) and t is the time in minutes, k_2 is the second order rate constant (g/mg.min), k_{IPD} is the intraparticle diffusion constant and C indicates the boundary layer thickness between the adsorbate and adsorbent, τ is the time needed to adsorb half of the equilibrium quantity in minutes; a is the time variation of rate constant; n is the fractional order of adsorption, q_e is the amount of adsorbate take up at equilibrium in mg/g and qt is the amount of adsorbate taken up at time, t in mg/g ,α and βare known as Elovich coefficients, which represent the initial adsorption rate (mg/g.min) and the desorption constant (g/mg), respectively.

Table S3. Comparison of adsorption capacities for TET on C300KT with literature. GS = graphene sponge, TiO_2(B) = monoclinic allotrope of TiO_2.

**Material**	**q_{max} (mg/g)**	**Ref.**
C300KT	30.0	This work

TiO_2(B) nanosheets@hydrochar composite	40.82 (TiO_2(B))<br>49.26 (entire composite with biochar)	1
Graphene oxide-TiO_2	63.69	2
Rice husk ash	8.37	3
Bamboo charcoal	22.70	4
Mesoporous BiOI Microspheres	28.35	5
Magnetic porous carbon	25.44	6

Table S4: Comparison of adsorption capacities for BPA on C300KT with literature. WC = wood charcoal, NCA = naphthalene carboxylic acid, Ti = titanium, Zr = zirconium, N/A= not available.

**Material**	**q_{max} (mg/g, % removal)**	**Ref.**
C300KT	23.0 (55)	This work
TiO_2-graphene hydrogel	q_{max} N/A (29.6)	7
TiO_2-WC-400	q_{max} N/A (60)	8
TiO_2-P25<br>P25-NCA<br>$Ti_{0.5}Zr_{0.5}O_2$-NCA	4.1 (TiO_2-P25)<br>7.5 (P25-NCA)<br>22.4 ($Ti_{0.5}Zr_{0.5}O_2$-NCA)	9
TiO_2-based organic-inorganic hybrid material	22.4	9
Graphite oxide	17.27	10
Monolayer cetylpyridinium bromide-modified natural zeolite	29.6	11

Tithonia diversifolia AC	15.69	12

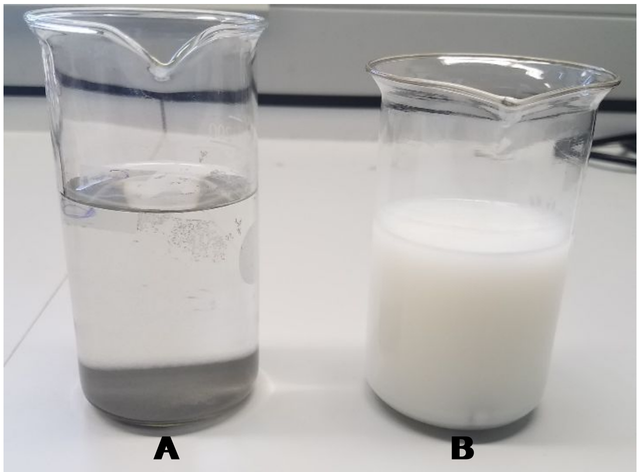

Figure S3: Comparison between the separation ability of a) C300KT and b) unmodified TiO_2 NPs in aqueous media after approx. an hour. Clearly, C300KT settles rather effectively while the titania NPs remain in the dispersions and are difficult to separate.

References

1. Mengting, Z.; Kurniawan, T. A.; Avtar, R.; Othman, M. H. D.; Ouyang, T.; Yujia, H.; Xueting, Z.; Setiadi, T.; Iswanto, I., Applicability of TiO2 (B) nanosheets@ hydrochar composites for adsorption of tetracycline (TC) from contaminated water. *Journal of hazardous materials* **2021,** *405*, 123999.

2. Wang, J.; Liu, R.; Yin, X., Adsorptive removal of tetracycline on graphene oxide loaded with titanium dioxide composites and photocatalytic regeneration of the adsorbents. *Journal of Chemical & Engineering Data* **2018,** *63* (2), 409-416.

3. Chen, Y.; Wang, F.; Duan, L.; Yang, H.; Gao, J., Tetracycline adsorption onto rice husk ash, an agricultural waste: Its kinetic and thermodynamic studies. *Journal of Molecular Liquids* **2016,** *222*, 487-494.

4. Liao, P.; Zhan, Z.; Dai, J.; Wu, X.; Zhang, W.; Wang, K.; Yuan, S., Adsorption of tetracycline and chloramphenicol in aqueous solutions by bamboo charcoal: a batch and fixed-bed column study. *Chemical engineering journal* **2013,** *228*, 496-505.

5. Hao, R.; Xiao, X.; Zuo, X.; Nan, J.; Zhang, W., Efficient adsorption and visible-light photocatalytic degradation of tetracycline hydrochloride using mesoporous BiOI microspheres. *Journal of hazardous materials* **2012,** *209*, 137-145.

6. Zhu, X.; Liu, Y.; Qian, F.; Zhou, C.; Zhang, S.; Chen, J., Preparation of magnetic porous carbon from waste hydrochar by simultaneous activation and magnetization for tetracycline removal. *Bioresource technology* **2014,** *154*, 209-214.

7. Zhang, Y.; Cui, W.; An, W.; Liu, L.; Liang, Y.; Zhu, Y., Combination of photoelectrocatalysis and adsorption for removal of bisphenol A over TiO2-graphene hydrogel with 3D network structure. *Applied Catalysis B: Environmental* **2018,** *221*, 36-46.
8. Luo, L.; Yang, Y.; Xiao, M.; Bian, L.; Yuan, B.; Liu, Y.; Jiang, F.; Pan, X., A novel biotemplated synthesis of TiO2/wood charcoal composites for synergistic removal of bisphenol A by adsorption and photocatalytic degradation. *Chemical Engineering Journal* **2015,** *262*, 1275-1283.
9. Suzuki, U.; Kameda, T.; Kumagai, S.; Saito, Y.; Yoshioka, T., Adsorption of bisphenol A by TiO2-based organic–inorganic hybrid materials. *Journal of Water Process Engineering* **2022,** *49*, 103081.
10. Bele, S.; Samanidou, V.; Deliyanni, E., Effect of the reduction degree of graphene oxide on the adsorption of Bisphenol A. *Chemical Engineering Research and Design* **2016,** *109*, 573-585.
11. Li, J.; Zhan, Y.; Lin, J.; Jiang, A.; Xi, W., Removal of bisphenol A from aqueous solution using cetylpyridinium bromide (CPB)-modified natural zeolites as adsorbents. *Environmental Earth Sciences* **2014,** *72* (10), 3969-3980.
12. Supong, A.; Bhomick, P. C.; Baruah, M.; Pongener, C.; Sinha, U. B.; Sinha, D., Adsorptive removal of Bisphenol A by biomass activated carbon and insights into the adsorption mechanism through density functional theory calculations. *Sustainable Chemistry and Pharmacy* **2019,** *13*, 100159.
